# The RNA thermometer motif ROSE-G regulates ABC transporter gene expression in bacteria

**DOI:** 10.1016/j.jbc.2025.111119

**Published:** 2025-12-30

**Authors:** Madelyn N. Mills, Gabriel Pacheo, Alina Y. Tong, Elisha L. Tong, Michael A. Hannani, Lana Heganovic, Kiana Fleary, Samantha N. Shaffer, Mallika S. Vairavan, Adrian R. Ferré-D’Amaré, Luiz F.M. Passalacqua, Michael M. Abdelsayed

**Affiliations:** 1 Department of Biology, California State University Northridge, Northridge, California, USA; 2 Department of Biology, California Lutheran University, Thousand Oaks, California, USA; 3 Department of Microbiology and Cell Science, IFAS, University of Florida, Gainesville, Florida, USA; 4 Laboratory of Nucleic Acids, National Heart, Lung, and Blood Institute, National Institutes of Health, Bethesda, Maryland, USA; 5 Department of Biochemistry and Molecular Biology, College of Medicine, University of Florida, Gainesville, Florida, USA

**Keywords:** RNA, ABC transporter, RNA structure, RNA thermometer, gene regulation, translation regulation, noncoding RNA, heat stress

## Abstract

RNA thermometers are temperature-sensing non-coding RNAs that regulate the expression of downstream genes. We previously reported that a well-characterized RNA thermometer, the ROSE-like element (repression of heat shock gene expression), is broadly distributed upstream of ATP-binding cassette (ABC) transporter genes in bacteria. ABC transporters are a superfamily of transmembrane proteins that harness ATP hydrolysis to facilitate the export and import of substrates across cellular membranes. Through structure-guided bioinformatics, we have now discovered a novel RNA motif, ROSE-G, that is closely related to the canonical ROSE-like motif. The newly identified ROSE-G motif is also widespread upstream of ABC transporter genes across diverse bacterial species. Structure probing, biochemistry, and cellular assays collectively indicate that this newly identified motif functions as an RNA thermometer. This study expands the known classes of RNA thermometers and further underscores the importance of RNA thermometers in the post-transcriptional regulation of ABC transporters in bacteria.

ABC transporters are a superfamily of transmembrane proteins that harness ATP hydrolysis to facilitate the export and import of an extensive array of substrates, such as metal ions, sugars, amino acids, peptides, iron-chelating compounds, vitamins, lipids, and drugs across cellular membranes (1, 2, 3). In bacteria, most of these transmembrane transporters share a characteristic structural arrangement consisting of at least four domains: two transmembrane domains (TMDs) and two ATP-binding domains (ABC domains) (2, 4). The ABC domains of these transporters exhibit a high degree of sequence conservation. In contrast, the TMDs exhibit substantial variability and are specialized for recognizing specific substrates, resulting in a diverse array of transporters represented by distinct operons and genes (2, 5). In prokaryotes, some ABC transporters have an additional fifth domain that contains a high-affinity binding protein for the substrate being transported (2, 5).

ABC transporters are ubiquitous across all domains of life and play essential roles in cellular processes such as nutrient acquisition, toxin efflux, and membrane homeostasis. These crucial functions contribute to important biological outcomes, including virulence, pathogenesis, and multidrug resistance (6, 7). In bacteria, ABC transporters are instrumental in responding to environmental conditions such as osmotic and oxidative stress, contributing to the survival of bacteria inside and outside the host environment (3, 4). Several studies suggest that RNA thermometers mediate heat stress–triggered translational upregulation of ABC transporter genes, indicative of a post-transcriptional mechanism that links temperature sensing to cell transport (8, 9, 10).

RNA thermometers are RNA elements found in the 5′ untranslated regions (5′-UTRs) of genes, where they regulate gene expression in response to changes in temperature (11, 12). To date, two conserved classes of RNA thermometers have been described. These regulate translation through a weak hydrogen-bonding network that drives temperature-dependent structural changes, which in turn confer access of ribosomes to the Shine-Dalgarno (SD) element. RNA thermometers in the 'fourU' class feature a terminal hairpin with at least four consecutive uracils base paired to the SD sequence, while those in the 'ROSE-like' (repression of heat shock gene expression) class contain a terminal hairpin with the SD sequence imperfectly paired to a conserved U(U/C)GCU (13, 14, 15, 16) (Fig. 1*A*). Increased temperature promotes destabilization of these motifs, followed by exposure of the SD for ribosomal binding, thereby driving increased gene expression (12, 17). Noncanonical G•U wobble base pairs or incomplete base-pairing resulting in bulged nucleotides in these motifs appear to contribute to their temperature responsiveness. Several candidate RNA thermometers lack these specific motifs, suggesting that RNA thermometers may encompass a diverse array of structural motifs. It remains uncertain whether these RNA thermometers represent isolated examples or belong to broader, yet unidentified classes.

**Figure 1:**
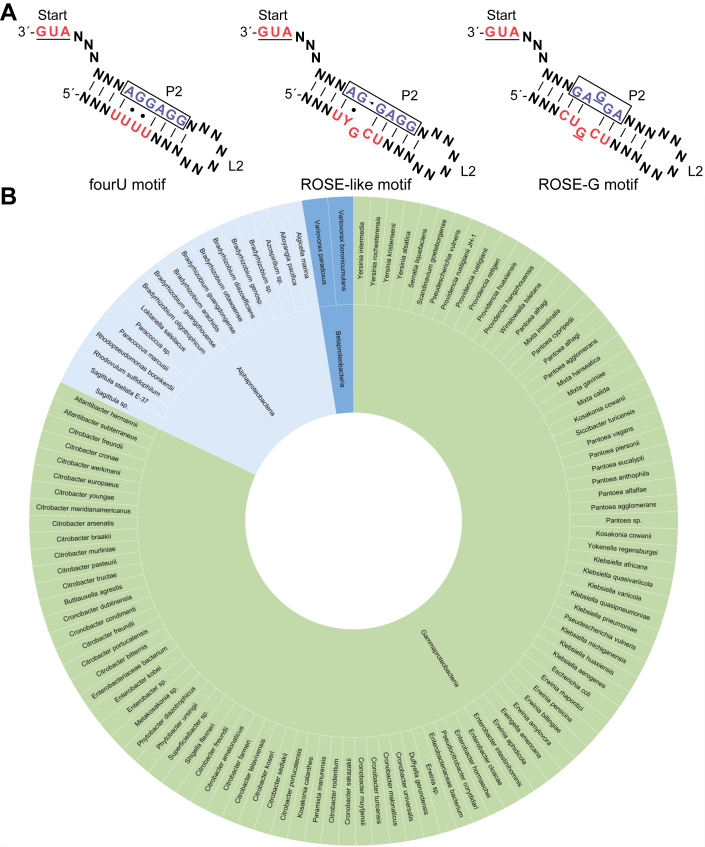
**Widespread occurrence of ROSE-like RNA thermometers upstream of ABC transporter genes in bacteria.***A*, secondary structure representation of the RNA thermometer classes fourU, ROSE-like, and ROSE-G. “N” represents any nucleotide, and these regions can vary in length. *Red sequences* are conserved and represent the sequence base-paired across from the SD sequence. Boxed nucleotides in *blue*, variable SD region. *B*, sunburst species distribution of ROSE-G RNA thermometer upstream of ABC transporter genes in bacteria. Inside tier of sunburst shows class distribution within the Pseudomonadota phylum.

We recently discovered that ROSE-like RNA thermometers are widespread upstream of ABC transporter genes across diverse bacterial species (10). In addition, two other ROSE-like RNA thermometers were predicted to occur upstream of ABC transporter genes (8), and a single non-ROSE-like RNA thermometer was validated in *Yersinia pseudotuberculosis* upstream of the *oppA* gene, which is a member of the ABC transporter family (9). Herein, we further performed iterative bioinformatic searches based on our previously published results. Unexpectedly, this revealed a new RNA thermometer motif, herein named 'ROSE-G', that is closely related in sequence to the ROSE-like motif. The newly identified ROSE-G features a different predicted structure compared to the ROSE-like motif. Its distinct sequence and structural features suggest that this motif represents a new class of RNA thermometers (Fig. 1*A*). This new thermometer motif is also highly conserved upstream of ABC transporter genes and may be more abundant than members of the ROSE-like motif.

## Results

We identified RNA thermometers containing the new ROSE-G motif by using Robo-Therm, our bioinformatics-based pipeline for the discovery of RNA thermometers (18). Robo-Therm employs the RNA motif search tool RNArobo (19), which allows users to fully handcraft and feature components of an RNA that are essential for its function and has been successfully implemented to discover several fourU and ROSE-like RNA thermometers. We expanded on our previously published results (10) by conducting several new iterative RNArobo searches using a generic ROSE-like motif as a template with highly permissive parameters, allowing sufficient variation to capture sequences that diverge from the original template motif. Additionally, we filtered these new results and our previous results of the putative ROSE-like RNA thermometers of ABC transporters through NCBI BLAST, applying multiple search configurations to enable the discovery of conserved variant sequences (Methods). Together, these approaches led to the discovery of the ROSE-G motif.

The newly identified ROSE-G motif features a different sequence and predicted structure compared with the ROSE-like motif. The ROSE-G motif contains a CUGCU sequence that pairs with the SD region in a characteristic pattern (Fig. 1*A*). Unlike the ROSE-like motif, ROSE-G forms a distinctive secondary structure where a bulged G within the motif is positioned opposite a corresponding bulged G in the SD region, an arrangement not observed in ROSE-like motifs (Fig. 1*A*). This motif has not yet been described in the literature, and its distinct sequence and structural features suggest it may have been missed by traditional sequence and structure-based discovery methods.

We have identified ROSE-G RNA thermometer candidates upstream of ABC transporter genes in 115 different bacterial species that belong to various families of the Pseudomonadota phylum of gram-negative bacteria (Figs. 1*B*, S1, and Table S1). These ROSE-G candidates occur upstream of different ABC transporter genes, including genes encoding the TMDs, ABC domains, and the additional high-affinity substrate binding protein domain. Many of these candidates appear upstream of the same or similar ABC transport genes (Table S1). Interestingly, several highly virulent and multidrug-resistant pathogens are present in our results (20). Additionally, our results also present many bacteria that are found in the human gut microbiome (21).

**Figure 2:**
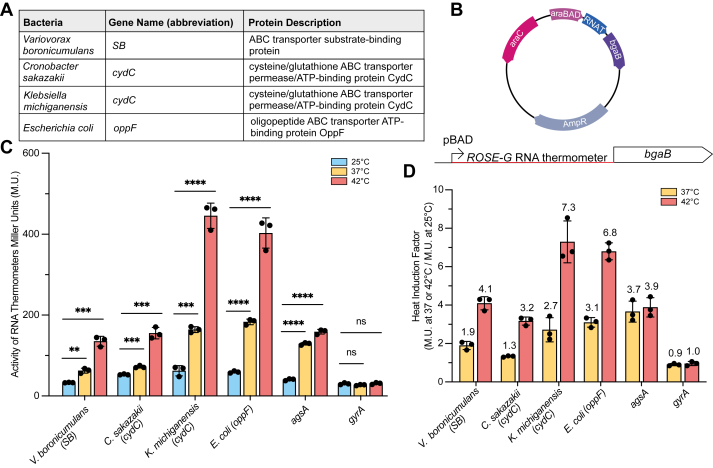
**ROSE-G RNA thermometer candidates upstream of ABC transporter genes.***A*, gene information of the four RNA thermometer candidates tested. *B*, map of the inducible reporter plasmid with the candidate thermometer placed directly upstream of the heat-stable β-galactosidase gene (*bgaB*) along with a schematic of *bgaB* fusions. *C*, expression in Miller Units (M.U.) of three ROSE-G RNA thermometers at 25, 37, and 42 °C compared to a positive control (*agsA* RNA thermometer) and a negative control (DNA gyrase - *gyrA*). (mean ± s.d., n = 3 biological replicates). For all four candidates, translation was significantly greater at 42 than at 25 °C with ∗∗∗ (*p* < 0.001), and ∗∗∗∗ (*p* < 0.0001); Student’s two-tailed *t* test. *D*, heat induction factor of all four candidates tested. Heat induction factor [activity in Miller Units (M.U.) from *panel C* at 37 °C/25 °C or 42 °C/25 °C] indicated on top of each bar. The *agsA* RNA thermometer is a positive control, and DNA gyrase (*gyrA*) is a negative control (mean ± standard deviation; n = 3 biological replicates).

We analyzed ROSE-G sequences based on secondary structure predictions (22) and selected four candidate RNA thermometers for further analysis. These are located upstream of three different ABC transporter genes: the substrate-binding subunit of an amino acid ABC transporter in *Variovorax boronicumulans* (23), the *cydC* gene, which encodes a subunit of the heterodimeric heme ABC transporter CydDC (24) in *Cronobacter sakazakii* and *Klebsiella michiganensis*, and the *oppF* gene, which encodes an ATP-binding subunit of the oligopeptide ABC transporter in *Escherichia coli* (25) (Figs. 2*A* and S2). Given that the ROSE-G element is highly conserved upstream of several genes with only minor variations, we sought to determine whether these differences impact regulatory activity. We tested two candidates upstream of *cydC* that each contain the ROSE-G motif but show small differences in sequence and predicted secondary structure (Fig. S2).

To determine the thermoregulatory activity of these four candidate sequences, we used a reporter system containing the 5′-UTR of candidate genes fused upstream of a heat-stable β-galactosidase (*bgaB*) (26) (Fig. 2*B*). These fusions were placed downstream of an arabinose-inducible promoter for temperature-independent control of transcription. Gene expression was tested in *E. coli* containing the 5′-UTR-*bgaB* fusions at 25, 37, and 42 °C. β-galactosidase activity was measured for each temperature, and heat induction profiles were calculated for each RNA thermometer [activity in Miller Units (M.U.) at 37 °C/25 °C or 42 °C/25 °C] (27) (Fig. 2, *C* and *D*). *bgaB* fusions containing the extensively characterized *agsA* RNA thermometer were used as a positive control, and the 5′-UTR DNA gyrase gene (*gyrA*), which is not thermally regulated, was used as a negative control (15). Heat shock of cells expressing 5′-UTR *V. boronicumulans bgaB* fusions resulted in a heat induction factor of 4.1-fold at 42 °C, consistent with the 5′-UTR of the *V. boronicumulans* substrate-binding subunit gene performing as an RNA thermometer, for which further evidence is provided below. Both *cydC* candidates displayed thermoregulatory activity, with 3.2 and 7.3- fold heat induction at 42 °C for *C*. *sakazakii and K. michiganensis*, respectively, indicating the ROSE-G can function as a thermometer in distinct sequence contexts. The *E. coli oppF* candidate also showed thermoregulatory function with a 6.8-fold heat induction at 42 °C, validating thermometer activity. Together, these results show that the ROSE-G motif remains thermoregulatory despite overall sequence variation, supporting its recognition as a new class of RNA thermometers.

**Figure 3:**
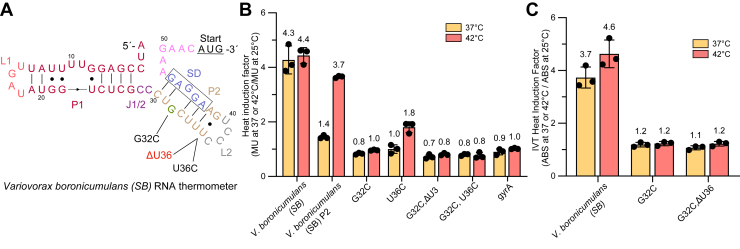
**Biochemical investigation of the *SB* ROSE-G RNA thermometer from *Variovorax boronicumulans*.***A*, predicted secondary structure of the tested ROSE-G RNA thermometer found upstream of the *SB* gene in *Variovorax boronicumulans*. Mutations tested for thermoregulation activity are depicted. Shine-Dalgarno sequence is boxed. *B*, heat induction factor of wild-type *V. boronicumulans SB* RNA thermometer, *P2* alone, mutants, and DNA gyrase (*gyrA*) negative control (mean ± standard deviation; n = 3 biological replicates). Heat induction factor [activity in Miller Units (M.U.) at 37 °C/25 °C or 42 °C/25 °C] indicated at the top of each bar. Gene expression of the *SB* wild-type UTR was significantly greater from expression of *SB* mutants at 42 °C and ranged from ∗∗∗ (*p* < 0.001) to ∗∗∗∗ (*p* < 0.0001); Student’s two-tailed *t* test. For individual Miller units and *p* values, refer to Fig. S3. *C, in vitro* translation heat induction factor levels *SB* RNA thermometer and two mutants by measuring 420 nm ABS (absorbance) at 37 and 42 °C compared to 25 °C. Translation was significantly greater at 42 °C than at 25 °C for the wild-type with ∗∗∗∗ (*p* < 0.0001); Student’s two tailed *t* test. For individual absorbance levels and *p* values, refer to Fig. S4 (mean ± standard deviation; n = 3 three technical replicates).

From our validated RNA thermometers, we focused on the ROSE-G RNA thermometer that occurs in the 5′-UTR of the substrate binding (SB) subunit of an amino acid ABC transporter in *V. boronicumulans* (*SB* thermometer). To confirm that the *SB* thermometer sequence directly mediates heat-dependent induction, mutations were introduced to strengthen and stabilize base pairing within its ROSE-G–containing stem (Fig. 3*A*). A hallmark of the ROSE-G motif is a characteristic double bulge formed by a bulged G within the ROSE-G sequence (CUGCU) that aligns with a corresponding bulged G in the Shine-Dalgarno (SD) region. To assess the functional importance of this structural feature, the bulged G was replaced with a C (CUCGU), converting the double bulge into a canonical C–G base pair (G32C). This stabilizing mutation completely abolished thermoregulatory activity, indicating that the double-G bulge greatly contributes to the local instability required for temperature-dependent unfolding (Figs. 3*B* and S3).

To further evaluate the role of weak base pairing surrounding the motif, additional stabilizing substitutions were introduced (Figs. 3*B* and S3). The U36C and ΔU36 mutations converted a U•G wobble pair preceding the loop into a stronger C–G pair. Both mutations significantly reduced heat induction. Interestingly, U36C retained modest activity at 42 °C, suggesting that base-pairing instability within the core motif is more critical than peripheral nucleotides surrounding the motif for thermoregulation. Combined mutations (G32C, ΔU36 or G32C, U36C), which further increase stem stability through additional C–G pairs, eliminated thermoregulatory function. Next, we investigated whether the P2 stem of the *SB* thermometer can achieve thermoregulation by itself. The P2 stem is the terminal helix that contains the conserved ROSE-G motif. P2 alone exhibited a heat induction of ∼3.7-fold at 42 °C, comparable to the full-length wild-type, indicating that this region is sufficient for full thermoregulatory activity. Together, these mutations demonstrate that both the ROSE-G motif and surrounding nucleotides within the hairpin structure in the *SB* thermometer are critical for thermoregulation.

Although our reporter construct utilizes an arabinose-inducible promoter to ensure temperature-independent transcription, we next performed studies to isolate translational regulation, as RNA thermometer function is primarily mediated at the post-transcriptional level. To distinguish between transcriptional and translational regulation in the system, we adopted the PURExpress *in vitro* translation system that has been used to validate RNA thermometer activity *in vitro* (17, 28, 29, 30). RNA was generated by *in vitro* transcription of *V. boronicumulans SB-bgaB* fusions, treated with DNase to remove template DNA, and subsequently purified. Similar amounts of RNA were then translated using the PURExpress system at 25, 37, and 42 °C to assess expression differences driven solely by translational control. Reactions were incubated for 1 hour, after which β-galactosidase activity was measured. Heat induction resulted in approximately 3.7- and 4.6-fold increases in activity at 37 and 42 °C, respectively, for the wild-type *V. boronicumulans SB*–*bgaB* fusion, confirming that the 5′-UTR mediates temperature-dependent translational regulation (Figs. 3*C* and S4). Stabilizing mutations (G32C and G32C, ΔU36) abolished thermoregulatory activity in the *in vitro* translation assay, further verifying that temperature-dependent structural changes within the ROSE-G motif are responsible for translational control.

Because *in vitro* translation assays lack cellular RNA-binding proteins, ribonucleases, and differ in ionic environment (magnesium and salt concentrations), quantitative differences between *in vivo* and *in vitro* thermoregulation are expected. Consistent with this, the fold-change observed in the PURExpress system was higher than that measured in cells. Similar discrepancies have been reported previously, for example, one study noted that the baseline increase in gene expression from 30 to 42 °C was larger *in vitro* than *in vivo*, potentially due to the magnifying effect of the high-yield PURExpress system (17). Taken together, these considerations indicate that while the absolute magnitude of induction may differ between systems, the *in vitro* results demonstrate the temperature-dependent translational regulation mediated by the *V. boronicumulans* 5′-UTR. Although RNA thermometers are typically understood to function by modulating ribosome accessibility to the ribosome-binding site, we acknowledge that alternative mechanisms, such as ribosome stalling, could produce similar temperature-dependent functionality. More mechanistically resolved approaches, including ribosome profiling, toeprinting, or related assays, could be employed in future studies to further elucidate whether ribosome binding alone accounts for the observed thermoregulation or whether additional translational processes contribute.

**Figure 4:**
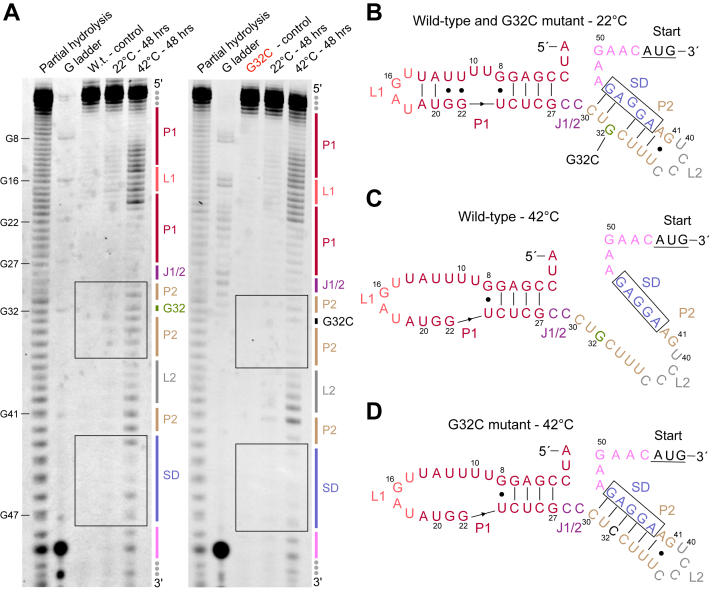
**Structural investigations of the *V. boronicumulans* ROSE-G RNA thermometer.***A*, in-line probing of 3′-end labeled wild-type (*left*) and G32C mutant (*right*) *SB* RNA thermometer. ROSE-G and SD regions are boxed. Secondary structure features labeled with same colors as *B*, in-line probing was done in duplicate. See Fig. S5 for the duplicate and for quantification of the intensity of bands at the ROSE-G and SD regions. *B*, secondary structure representation of the of wild-type and G32C mutant at 22 °C. *C*, secondary structure representation of the of wild-type at 22 °C. *D*, secondary structure representation of the of G32C mutant at 42 °C.

To explore the structure–function relationship underlying temperature sensing by the *SB* RNA thermometer, we investigated how the ROSE-G motif undergoes temperature-driven conformational changes. The prevailing mechanism of RNA thermometer thermoregulation involves partial denaturation of the terminal hairpin, which unfolds in a zipper-like manner at higher temperatures (12). However, it has also been suggested that some localized base flexibility, rather than complete hairpin melting, may also contribute to the regulatory response. (17, 31). We directly compared the wild-type to the impaired mutant G32C, which converts the bulged Gs into the canonical C–G base pair, using in-line structure probing (Fig. 4*A*). In-line probing is a chemical RNA probing method that provides information regarding the RNA secondary structure by quantifying the spontaneous intramolecular cleavage of RNA, due to local backbone flexibility (32). At 22 °C, both the wild-type and the mutant exhibited limited cleavage, suggesting a stable conformation for both molecules (Figs. 4, *A* and *B* and S5). However, at 42 °C, the wild-type was considerably less stable than the mutant, particularly in the ROSE-G motif and the SD region, indicating that the RNA is unstructured in these regions (Figs. 4 and S5*A*). In addition, quantification of band intensity in the regions of the ROSE-G and SD showed a considerable reduction in cleavage on the more stable and non-functional RNA thermometer, G32C mutant (Fig. S5*B*). Interestingly, P1 partially melts at 42 °C for both wild-type and G32C mutant, making the L1 loop bigger and more pronounced. Overall, these results demonstrate that the conserved ROSE-G element undergoes temperature-dependent conformational changes through the opening of the SD region within the P2 stem–loop, which is necessary for thermoregulatory activity of the *SB* RNA thermometer.

## Discussion

ABC transporters are ubiquitous membrane proteins that mediate the transport of a wide variety of substrates essential for cellular metabolism and survival (1, 2, 3, 4, 5). In bacteria, ABC transporters play key roles in adapting to the environment, particularly during periods of nutrient limitation and host-imposed stress. Despite ABC transporters playing a critical role in bacterial stress responses, the extent to which heat stress influences transporter expression is still unclear (3, 4, 5). Previously, we used our pipeline Robo-Therm (18) demonstrating that ROSE-like RNA thermometers are widespread upstream of ABC transporter genes across diverse bacterial species (10). Additionally, two other ROSE-like and single non-ROSE-like RNA thermometer were reported upstream of ABC transporters (8, 9). Together, these findings suggest that ABC transporter genes are widely regulated by RNA thermometers.

Currently, only two classes of RNA thermometers are known to occur widely, the fourU and ROSE-like motifs, both of which have been repeatedly identified in different bacteria species and upstream of diverse genes. Several characterized RNA thermometers lack these specific motifs, suggesting that RNA thermometers may encompass a diverse array of structural motifs. We extended upon our prior work by applying RNArobo searches with intentionally permissive parameters to our previously annotated ROSE-like RNA thermometers. This approach enabled the identification of novel motifs that retain the potential to function as RNA thermometers while diverging sufficiently from the original template to represent distinct variants. The same strategy can be applied to other classes of RNA thermometers or regulatory RNAs to uncover new motif classes and broaden the landscape of functional RNA discovery.

Our structure predictions uncovered a novel RNA thermometer motif, ROSE-G, with distinct sequence and structural features, supporting its potential as a new motif and class of RNA thermometers. We have predicted ROSE-G RNA thermometer candidates upstream of ABC transporter genes in 115 different bacterial species that belong to various families of the Pseudomonadota phylum of gram-negative bacteria (Fig. 1*B* and Table S1). This contrasts with the phylogenetic breadth of the distribution of the 41 ROSE-like RNA thermometer candidates found upstream of ABC transporter genes, which were found in four different phyla of both gram-positive and gram-negative bacteria (10). The ROSE-G is likely related to the putative ROSE-like motif, which suggests that both diverged from a common ancestor. The ROSE-G motif has not yet been documented in the literature, and its distinct sequence and structural features suggest it may have been missed by traditional sequence and structure-based discovery methods.

We demonstrated temperature-dependent gene regulation by four different ROSE-G thermometers in the 5′-UTRs of three different ABC transporter genes across diverse bacterial species. Our biochemical and structural characterization of the *V. boronicumulans SB* thermometer suggests that thermoregulation occurs post-transcriptionally and is dependent on the ROSE-G motif. To validate that the ROSE-G thermometer sequence is directly responsible for the increase in heat induction, mutations were made to stabilize base pairing. Interestingly, a single mutation that changed the opposing G bulges to a C–G base pair completely eliminated thermometer activity, indicating that the double G bulge significantly contributes to temperature-dependent gene expression. A key difference between the ROSE-G and the ROSE-like motifs is the presence of C–G and U•G base pairs. While many ROSE-like thermometers present only a single C–G base pair and a single U•G wobble base pair, the ROSE-G motif presents two C–G base pairs and lacks a wobble base pair. The bulged G and the wobble base pair on ROSE-like domains play an important role in disrupting the base stacking and the helical twist of the stem, which results in a decrease of its melting temperature (10). This suggests that the presence of two opposing bulged Gs in the ROSE-G motif compensates for the lack of a wobble base pair and the additional stability of the extra C–G base pair.

Beyond the ROSE-G–SD interactions in the terminal SD-containing hairpin, other regions may play an important role in modulating the RNA thermometer functionality. For instance, loop 2 and its closing base pair may impact the melting of the RNA thermometer. In our study, the mutation U36C mutation alone (part of the loop 2 closing base pair) greatly reduces the RNA thermometer functionality, suggesting that the opening of loop 2 is also important. Notably, among several of our candidates, including in *V. boronicumulans*, loop 2 is a tetraloop. Tetraloops, particularly UNCG and GNRA families, and their closing base pair may confer greater thermodynamics stability (33). In our case, the loop 2 sequence is CCCU, being one mutation away from a UNCG tetraloop, suggesting that a single mutation in the loop itself could make the hairpin more stable and modulate the entire RNA thermometer. Moreover, if combined with a C–G closing base pair that greatly improves the thermodynamic stability of these tetraloops (33), this change could be even greater. Therefore, regions outside the SD and the ROSE-G motif may also play an important role in modulating the temperature responsiveness of RNA thermometers.

To further characterize the regulatory mechanism of the *SB* thermometer, we performed additional experiments to clarify the basis of its temperature responsiveness. *In vitro* assays demonstrated that the increase in gene expression observed at higher temperatures is regulated by translational control. Lastly, our structural studies at both 22 and 42 °C confirmed that temperature-dependent structural changes occurred as predicted, showing that the ROSE-G motif undergoes a structural change that locally denatures the stem and exposes the SD sequence. This study highlights that other RNA thermometer classes may exist and have gone undiscovered while expanding on the importance of RNA thermometers in the post-transcriptional regulation of ABC transporters. Our data demonstrates that RNA thermometers employ manifold strategies to yield temperature control of gene expression, and that the ROSE-G motif constitutes a novel, third class of RNA thermometers.

## Experimental procedures

### RNA motif search in genomic sequences

Bacterial genomic sequences were downloaded from the NIH National Library of Medicine – National Center for Biotechnology Information using the Nucleotide search (https://www.ncbi.nlm.nih.gov/nuccore/). RNArobo (19) was used to perform RNA motif searches. We conducted iterative searches beginning with the parameters used to discover ROSE-like thermometers upstream of ABC transporters (10). Through successive refinement, we designed a modified descriptor that returned candidate motifs which sufficiently differentiated from those identified in our previous searches. Using RNArobo and the descriptor below, we identified a new class of candidate RNA thermometers that we designate ROSE-G.

s1 h1 s2 h2 s3 h2′ s4 h1′ s5

s1 0 ∗∗∗

h1 0:0 ∗∗∗∗NT:RG∗∗∗∗

s2 0 G

h2 0:0 NU∗∗∗∗:∗∗∗∗RG

s3 0 NNN∗∗∗∗

s4 0 ∗

s5 0 ∗∗∗∗∗∗∗NNAUG.

To further ensure specificity for the ROSE-G variant and to exclude previously characterized ROSE-like elements, we developed a second, more restrictive descriptor. This refined search produced hits exclusively corresponding to the ROSE-G variant.:

s1 h1 s2 h2 s3 h2′ s4 h1′ s5

s1 0 ∗∗∗

h1 0:0 ∗∗∗∗CT:RG∗∗∗∗

s2 0 G

h2 0:0 NU∗∗∗∗:∗∗∗∗RG

s3 0 NNN∗∗∗∗

s4 0 G

s5 0 ∗∗∗∗∗∗∗NNAUG.

Results were manually curated and verified with Basic Local Alignment Search Tool (BLAST)—NIH National Library of Medicine—National Center for Biotechnology Information (https://blast.ncbi.nlm.nih.gov/). Each candidate sequence from our previous study (10) and new RNArobo results were evaluated using three BLAST algorithms: megablast, discontiguous megablast, and blastn. Running each candidate through this BLAST workflow enabled identification of both highly similar matches (megablast) and more divergent variants (discontiguous megablast and blastn), thereby maximizing recovery of all potential ROSE-G candidates.

### Accession numbers

**Table:** 

Bacteria	Gene	Accession Number
*Variovorax boronicumulans*	*JVX96_26690 (SB)*	CP027773.1
*Cronobacter sakazakii*	*cydC*	CP028974.1
*Klebsiella michiganensis*	*cydC*	CP084542.1
*Escherichia coli*	*oppF*	CP089930.1

Full sequences tested and predicted secondary structures in the supplemental (Fig. S2 and Table S2).

### Phylogenetic tree

Analysis for a phylogenetic tree comprised of 115 bacterial species containing the P2 stem in the ROSE-G thermometer (Table S1). Sequences were aligned *via* muscle alignment and constructed with the maximum likelihood method in MEGA X (34). Branch lengths reflect the amount of genetic change between taxa. The tree was visualized in iTOL (35).

### Plasmid construction

Plasmids were synthesized from VectorBuilder (VectorBuilder Inc). The 5′-UTR of thermometer candidates (Table S2) were placed directly upstream of a heat-stable β-galactosidase from *Bacillus stearothermophilus* (26) and driven by a pBAD promoter (pBAD: β-galactosidase). ATG (start codon) in the thermometer sequences replaces the first ATG of *bgaB*. Full vector sequence can be retrieved from the VectorBuilder database using each unique vector ID (https://en.vectorbuilder.com/design/retrieve.html). Vector information is listed below.

**Table:** 

Bacteria	Gene	VBID
*Variovorax boronicumulans*	*JVX96_26690*	VB241026–1059
*Cronobacter sakazakii*	*cydC*	VB241026–1046
*Klebsiella michiganensis*	*cydC*	VB241026–1047

NEBuilder HiFi DNA Assembly was used to insert *E. coli* and mutation sequences into the same plasmid backbone described above. NEBuilder HiFi DNA Assembly was performed according to the manufacturer's protocol. A previously described vector used to validate the *blyA* thermometer (20) (VectorBuilder ID: VB220225–1020jdm) was used as the backbone for plasmid construction. NEBuilder Assembly Tool 2.0 was used to design fragments. The mutant inserts tested in the β-galactosidase assay (Table S2) were designed with the following complementary flanking sequences to the VB220225-1020jdm plasmid:

5′- ATACCCGTTTTTTGGGCTAA - Sequences for β-galactosidase assay (Table S2) - AATGTGTTATCCTCAATTTG -3′

### β-Galactosidase assays

*E. coli* DH5α cells carrying *bgaB* plasmids were grown overnight at 25 °C in LB broth plus 100 μg/ml ampicillin. Overnight cultures were diluted in LB broth plus 100 μg/ml ampicillin to an optical density at 600 nm (OD600) of 0.1, and then grown at 25 °C to an OD600 of 0.3 to 0.5. Transcription was induced with 0.01% (w/v) arabinose addition, then they were split and incubated at 25, 37, or 42 °C. After 60 min, 500 μl samples were taken, OD600 was measured, and samples were used for β-galactosidase assays as previously described (10, 16, 36) with the following modifications. Three 20 μl samples of culture were added to 80 μl of permeabilization solution (0.8 mg/ml hexadecyltrimethylammonium bromide, 0.4 mg/ml sodium deoxycholate, 100 mM Na_2_HPO_4_, 20 mM KCl, 2 mM MgSO_4_, and 5.4 μl/ml β-mercaptoethanol). After a 30-min incubation at 30 °C, 600 μl of substrate solution (60 mM Na_2_HPO_4_, 40 mM NaH_2_PO_4_, 1 mg/ml o-nitrophenyl-β-D-Galactoside (ONPG), 2.7 μl/ml β-mercaptoethanol) was added. The reactions were incubated at 55 °C for 90 min. The addition of 700 μl of 1 M Na_2_CO_3_ terminated the reactions to be prepared for absorbance readings. Assays were performed in triplicate. Heat induction factor is calculated by dividing the expression in Miller Units at 37 or 42 °C by the expression at 25 °C.

### *In vitro* translation assay

PCR amplification of DNA templates was performed with Phusion High-Fidelity DNA Polymerase (New England Biolabs). Each 20 μl reaction contained 10 μl 2 × Phusion High-Fidelity Master Mix, 2 μl T7 forward primer (10 μM), 2 μl *bgaB* reverse primer (10 μM), 2 μl plasmid DNA (1000× dilution), and 4 μl nuclease-free water. Thermal cycling included an initial denaturation at 95 °C for 30 s, followed by 35 cycles of 95 °C for 30 s, 55 °C for 30 s, and 72 °C for 2 min, with a final 5 min extension at 72 °C. PCR products were stored at −20 °C until gel verification or purification. Amplification conditions followed the manufacturer’s instructions.

PCR products were purified using the DNA Clean & Concentrator-5 kit (Zymo Research) according to the manufacturer’s protocol. For each PCR reaction, DNA binding Bugger was added at a 5:1 ratio to the sample volume, mixed, and transferred to a Zymo-Spin column. Columns were centrifuged at 10,000 to 16,000*g* for 30 s, washed twice with 200 μl DNA Wash Buffer, and eluted with 20 μl of nuclease-free water. DNA concentrations were measured, and purified products were stored at −20 °C or used directly for *in vitro* transcription.

*In vitro* transcription was performed with MEGAscript T7 Transcription Kit (Thermo Fisher Scientific). Purified DNA with T7 promoter-tagged *bgaB* inserts were used as DNA templates, following manufacturer protocols. Each 20 μl reaction included 2 μl of 40 mM (10 mM each) ATP, CTP, GTP, and UTP; 2 μl of 10× reaction buffer, 2 μl enzyme mix; and 0.1 to 1 μg of DNA, with nuclease-free water added to bring the final volume to 20 μl. Reactions were incubated at 37 °C for at least 4 h, followed by DNase I treatments to remove any residual DNA. RNAs were purified *via* lithium chloride (LiCl) precipitation according to the manufacturer's instructions.

*In vitro* translation was performed using the PURExpress *In Vitro* Protein Synthesis Kit according to manufacturer’s instructions. Either ∼1 ug (wild-type) ∼3 ug (mutants) of RNA was added into a 25 μl reaction and translated for an hour at the desired temperature. After translation, 5 μl of 1 mg/ml ONPG was added, and reactions were incubated at 55 °C for 90 min. Reactions were diluted with 150 μl of nuclease-free water then absorbance at 420 nm was measured.

### In-line structure probing

RNAs were transcribed from PCR templates (purchased from IDT) with T7 RNA polymerase, and purified by denaturing gel electrophoresis (10% polyacrylamide, 29:1 acrylamide: bisacrylamide; 1 × TBE, 8 M urea). After ultraviolet shadowing and excision from gels, RNAs were eluted from the gel into 300 μl of 300 mM KCl and precipitated by adding 700 μl of 100% ethanol at −20 °C. RNAs were resuspended in water. Next, RNAs were 3′ end labeled using T4 RNA Ligase 1 (New England Biolabs) and pCp-AZDye488 (Jena Bioscience) according to standard procedure from NEB. Labeled RNAs were purified using standard phenol-chloroform extraction followed by ethanol precipitation. Purified 3′ end-labeled RNAs were resuspended in water.

The 3′ end labeled RNAs (∼1 μM) were incubated for 48 h at 22 or 42 °C in buffer containing 140 mM KCl, 20 mM HEPES (pH 8.5), and 1 mM MgCl2. Reactions were quenched in a solution of 95% Formamide and 25 mM EDTA. The partially hydrolyzed RNAs were resolved on a 10% denaturing PAGE gel. The resolved gel was scanned on a GE Typhoon imager. The sequences in the degradation pattern were assigned by running ribonuclease T1 digestion and alkaline hydrolysis in parallel lanes, as previously reported (32). This experiment was performed in duplicate.

For band intensity quantification, ImageJ (37) was used. In summary, intensities of ROSE-G and Shine-Dalgarno regions at 42 °C were measured and normalized to the loop 1 and A48 + A49 regions (these control regions do not change between the wild-type and mutant experiments). Results were plotted using Microsoft Excel.

## Data availability

The data underlying this article are available in the article and in its online Supporting Information material.

## Supporting information

This article contains supporting information.

## Conflict of interest

The authors declare that they do not have any conflicts of interest with the content of this article.
