# Supplementary material for: The RNA thermometer motif ROSE-G regulates ABC transporter gene expression in bacteria
Source: J Biol Chem. 2025 Dec 30;302(2):111119. doi: 10.1016/j.jbc.2025.111119 (PMC12857270; doi:10.1016/j.jbc.2025.111119)
Supplement: Supporting information [file mmc1.docx]

The RNA thermometer motif ROSE-G regulates ABC transporter gene expression in bacteria

**SUPPORTING INFORMATION**

**Supporting Figures S1–S5 with legends and Tables S1–S2 with legends**

**Supporting Figure S1**


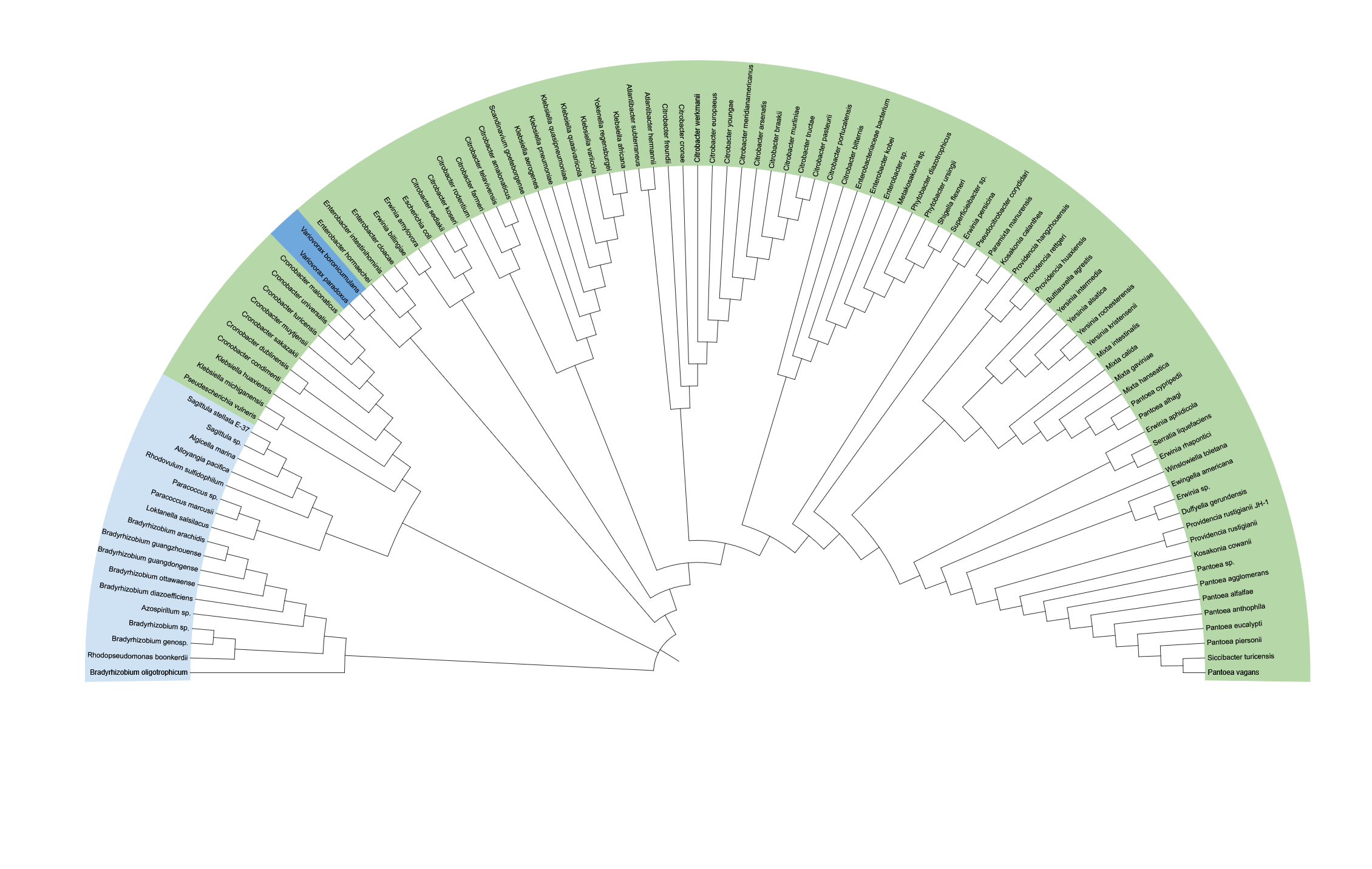


Gammaproteobacteria

Alphaproteobacteria

Betaproteobacteria

**Figure S1. ROSE-G phylogenetic tree.** Sequence relationship of 115 bacterial species that contain a predicted ROSE-G RNA thermometer sequence upstream of ABC transporter genes.

Gammaproteobacteria

Alphaproteobacteria

Betaproteobacteria

**Supporting Figure S2**

*Variovorax boronicumulans* (SB):


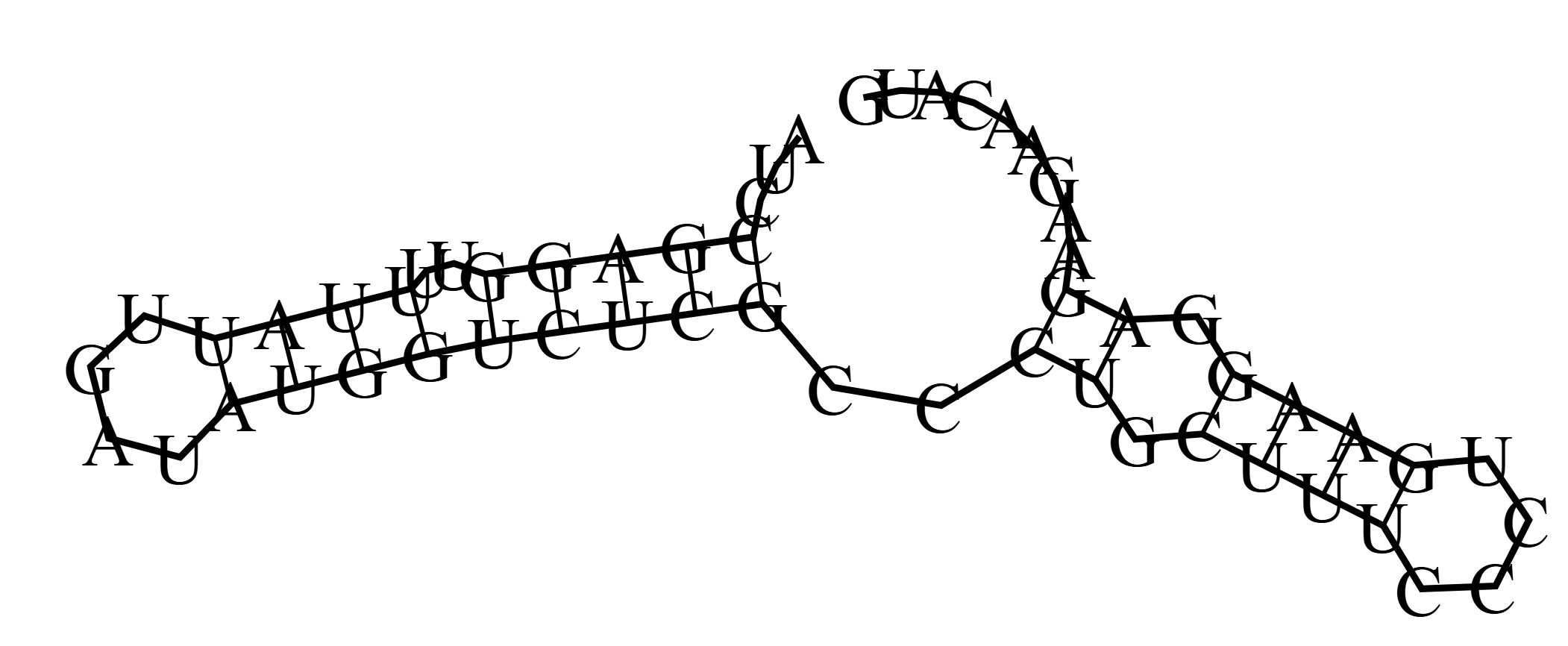


*Cronobacter sakazakii (cycD):*

*
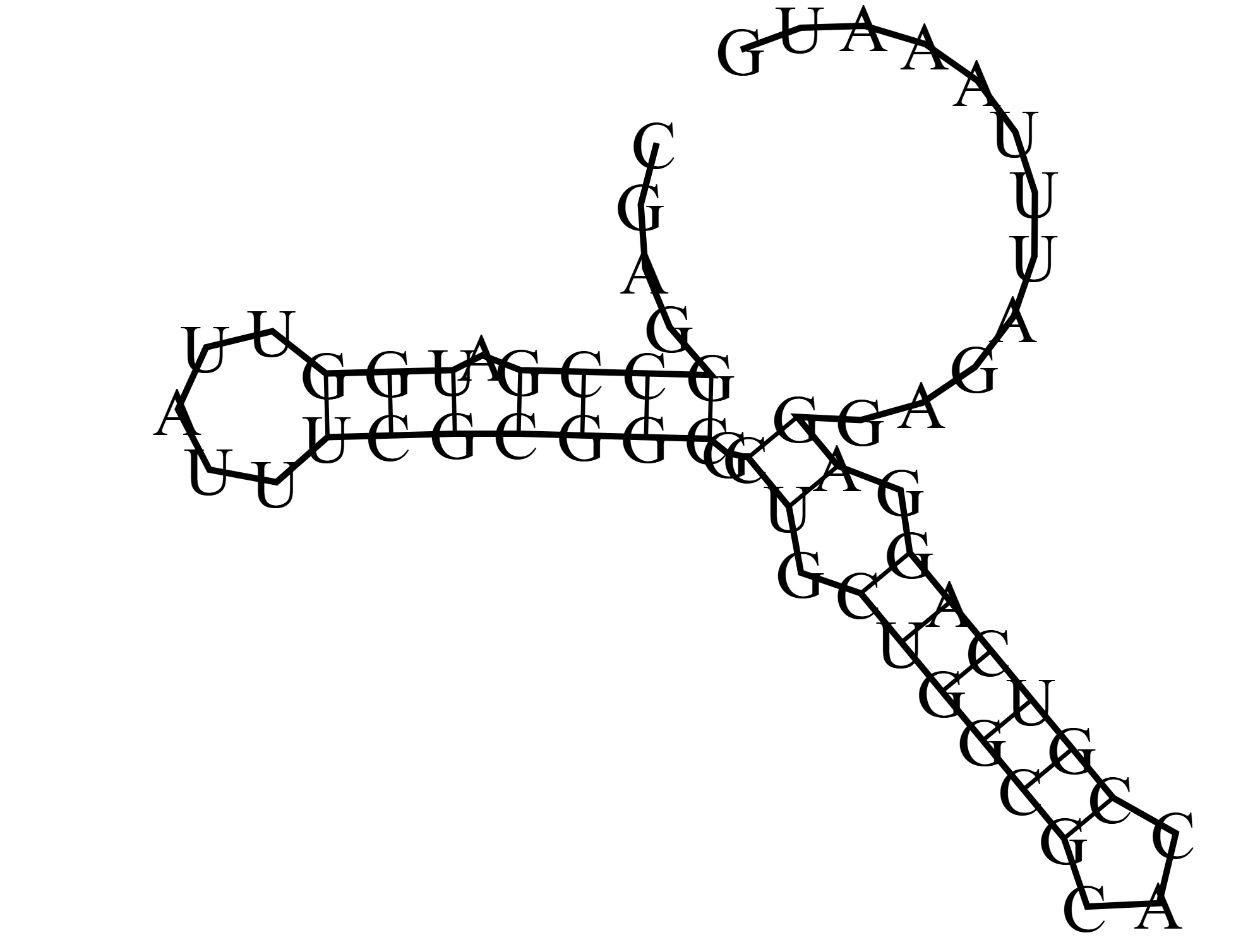
*

*Klebsiella michiganensis (cycD)*

*
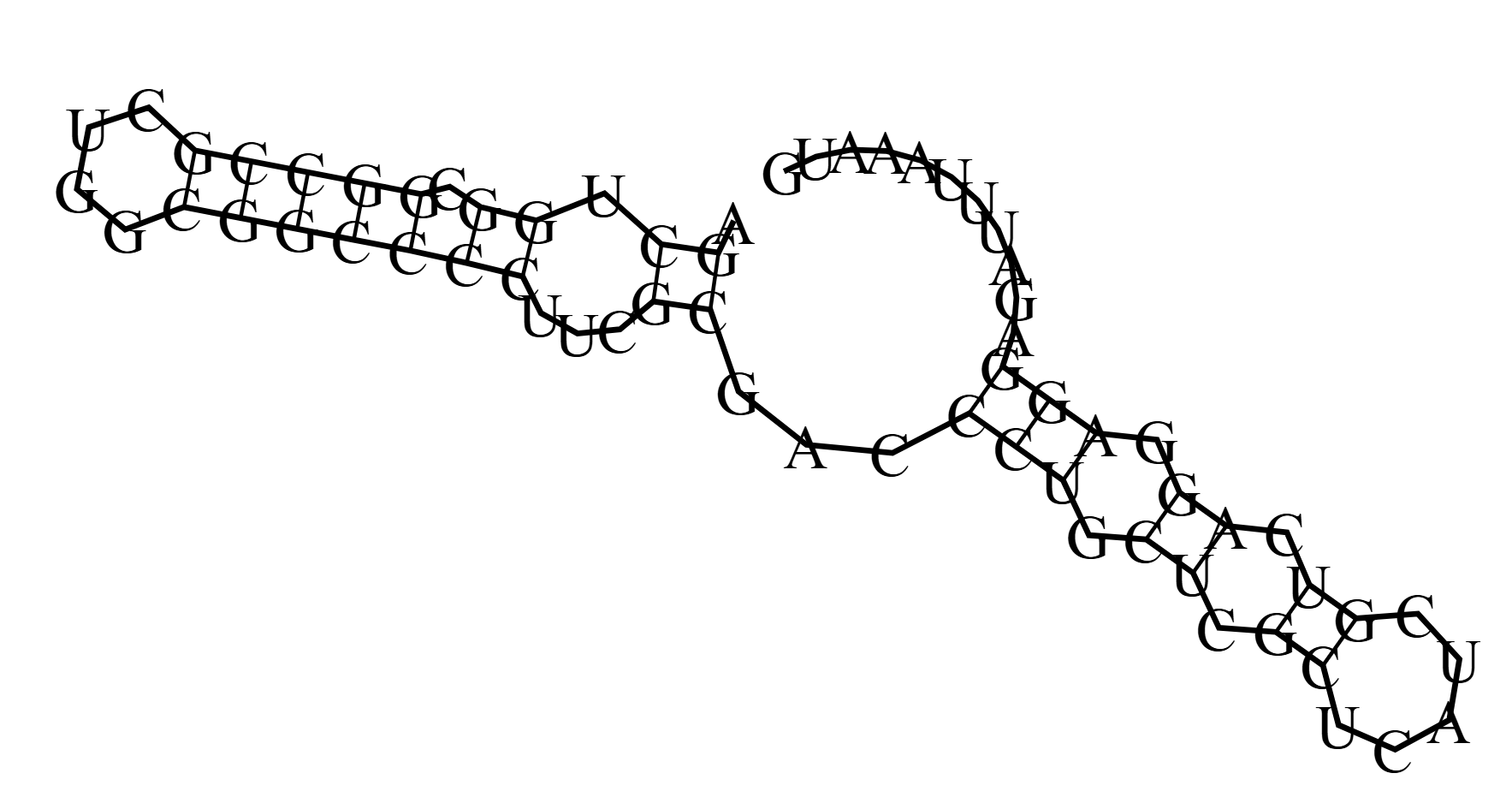
*

*Escherichia coli (oppF)*

**
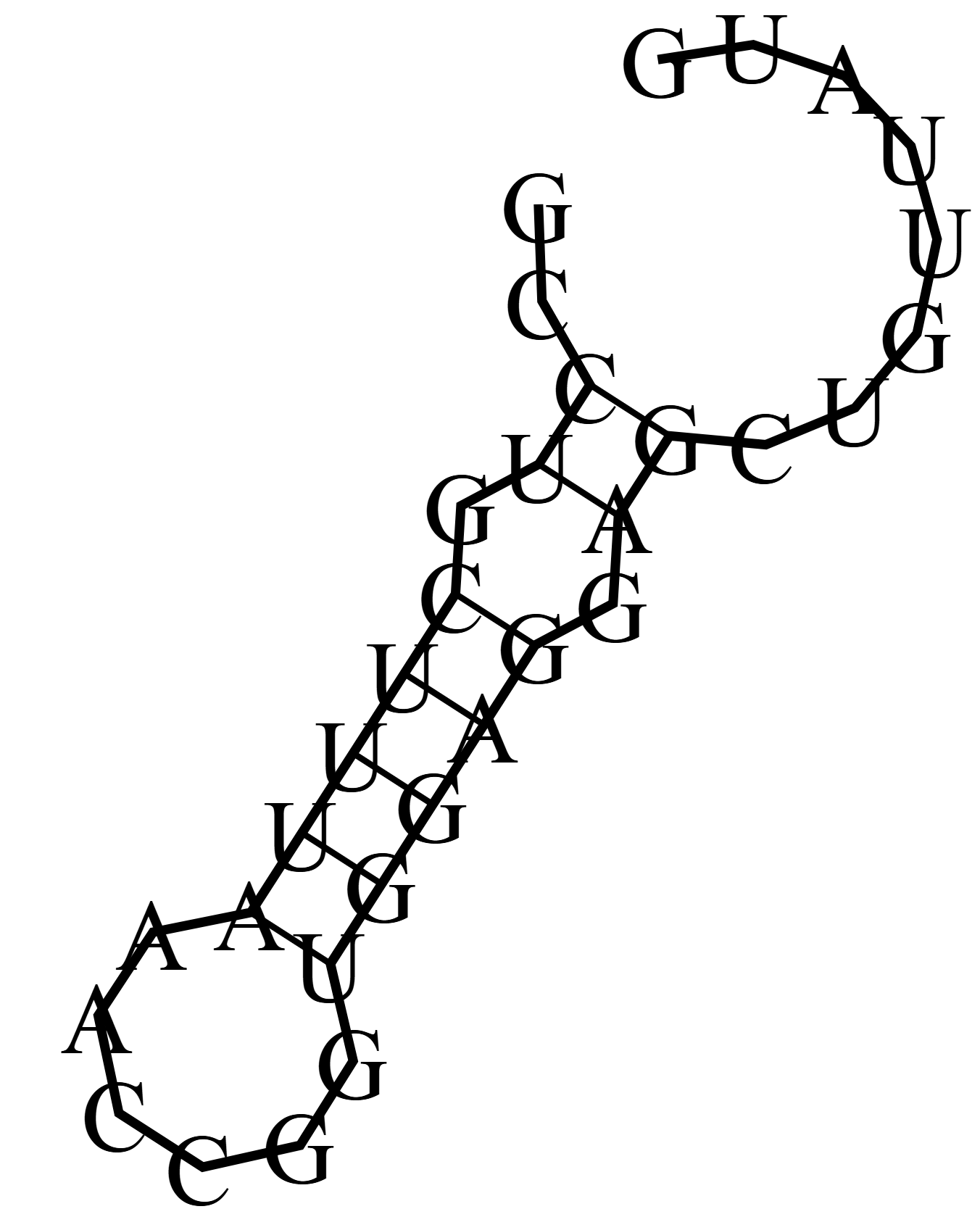
**

**Figure S2. Predicted secondary structures of tested ROSE-G RNA thermometers.** Boxed nucleotides, ROSE-G and SD elements.

**Supporting Figure S3**

**
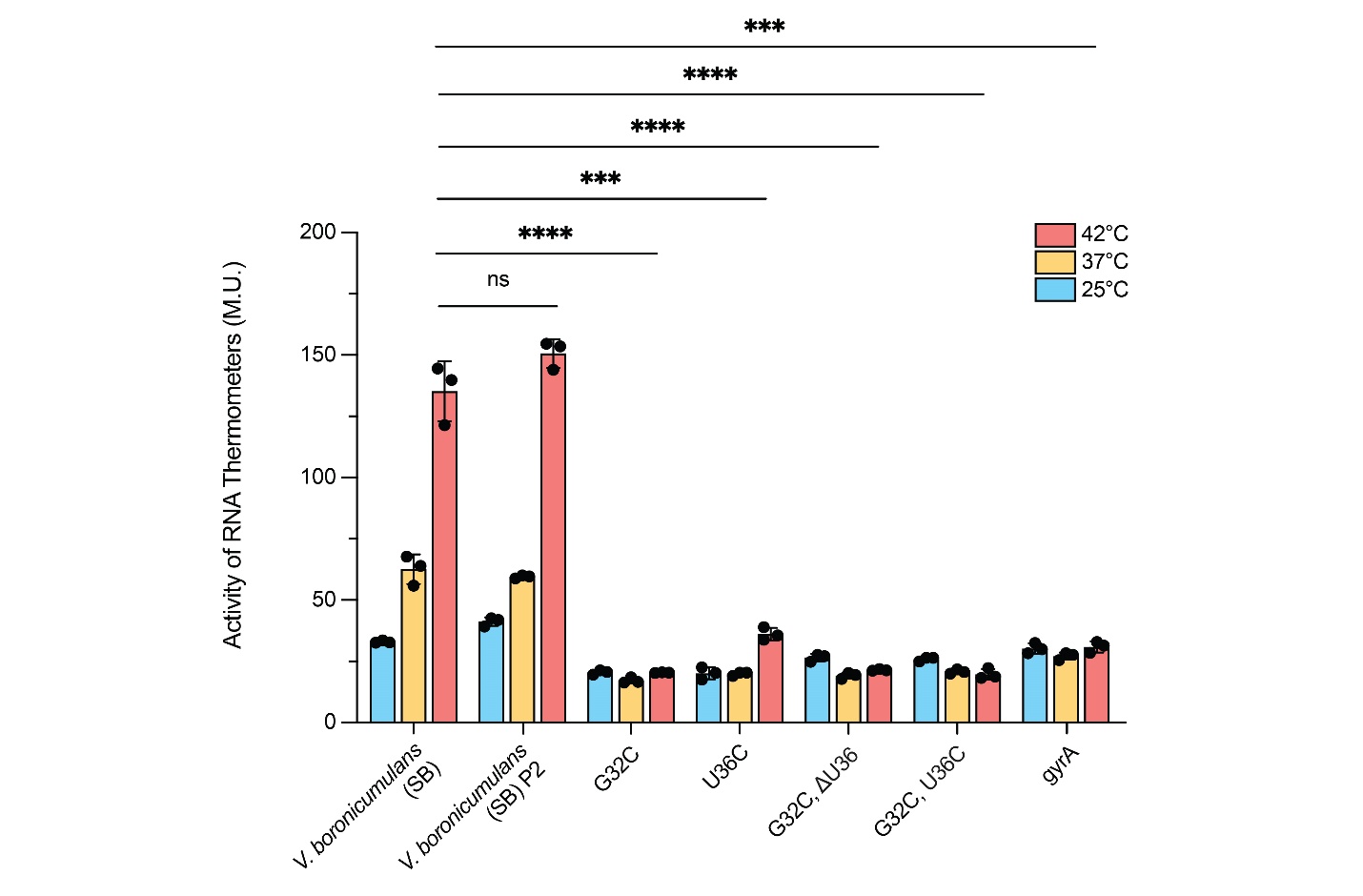
**

**Figure S3. Thermoregulatory activity of mutants.** Expression in Miller Units (M.U.) of *SB* mutants at 25, 37, and 42 °C compared to *SB* wild-type and a negative control (DNA gyrase - *gyrA*). (mean ± s.d., n = 3 biological replicates). Expression of the *V. boronicumulans (SB)* wild-type UTR was significantly different from translation of *SB* mutants at 42 °C with *** (*p* < 0.001), and **** (*p* < 0.0001); Student’s two-tailed *t* test.

**Supporting Figure S4**


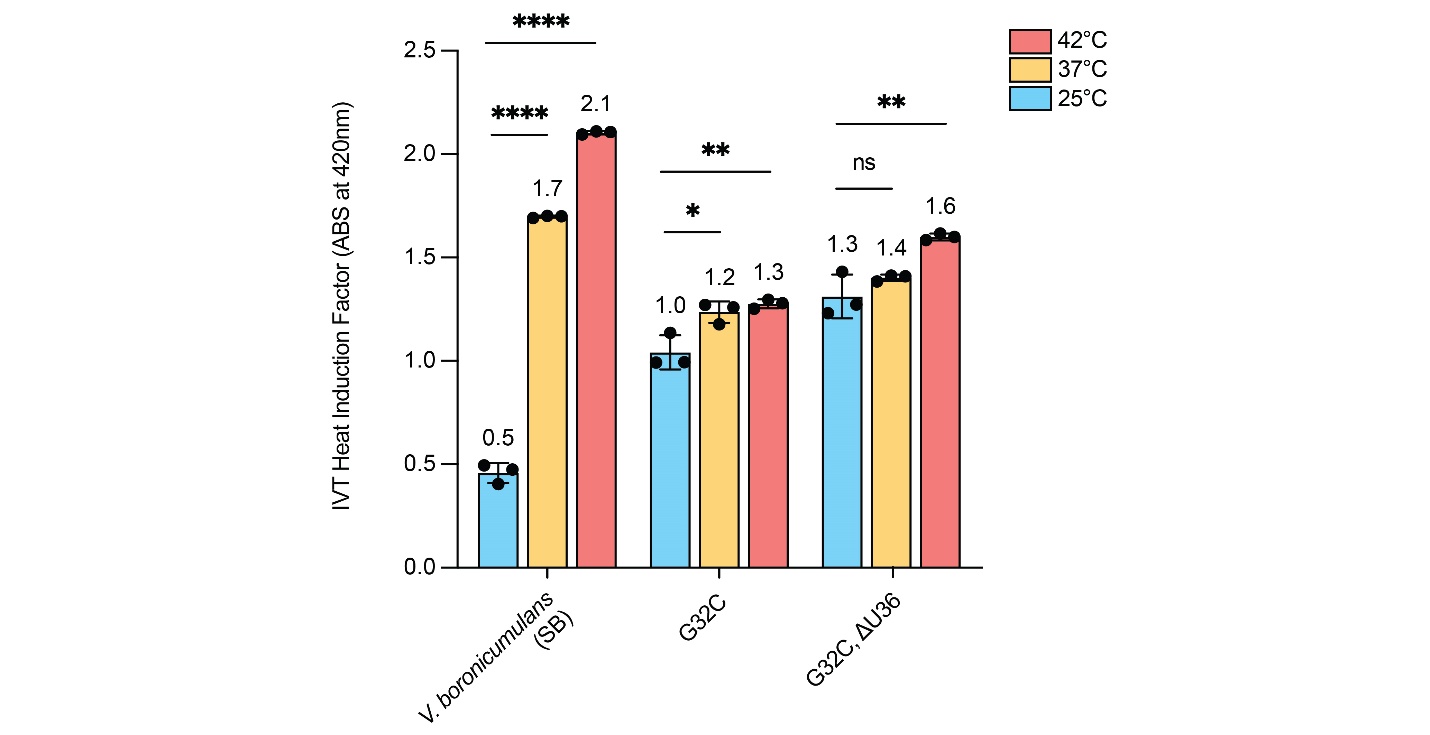


**Figure S4. *In vitro* translation heat induction factor.** *In vitro* translation (IVT) activity of *V. boronicumulans* (SB) wild-type RNA thermometer and corresponding mutants at 25, 37, and 42 °C (absorbance at 420 nm). (mean ± s.d., n = 3 technical replicates). IVT of the *V. boronicumulans (SB)* wild-type UTR was significantly greater 42 °C than at 25 °C with **** (*p* < 0.0001); Student’s two-tailed *t* test.


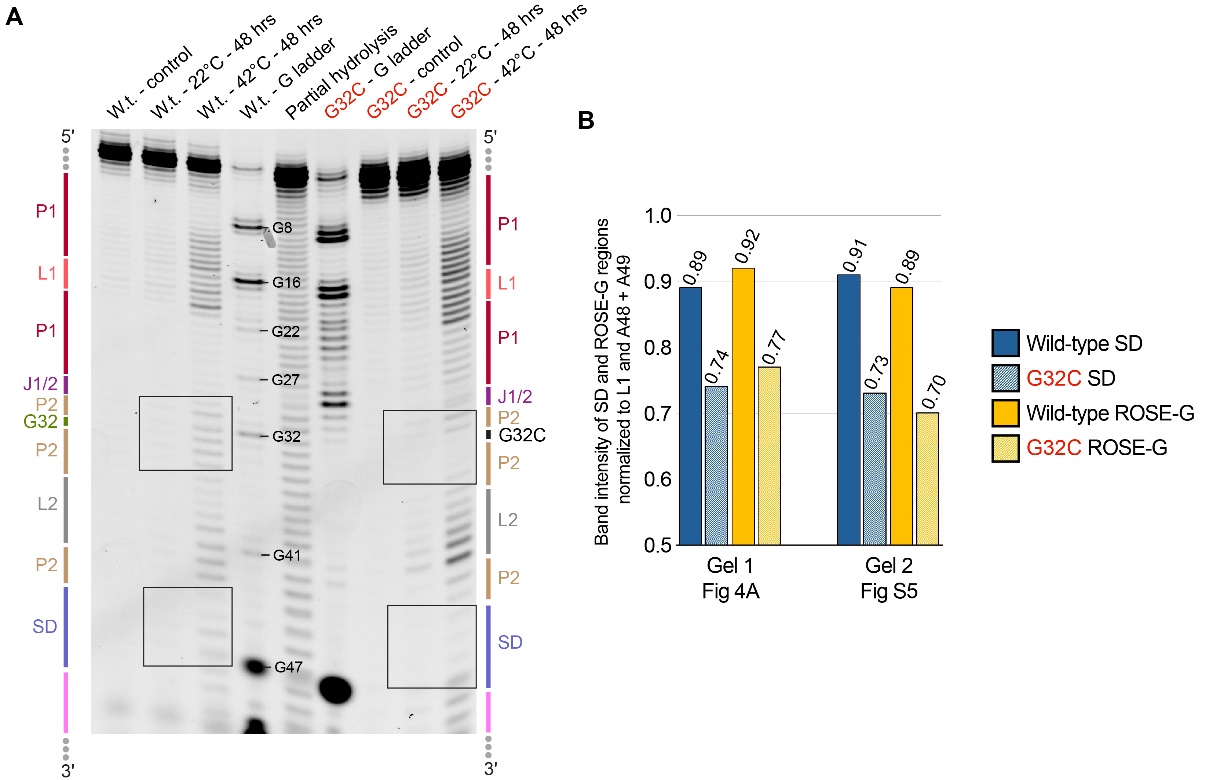
**Supporting Figure S5**

**Figure S5. Structural investigations of the *V. boronicumulans*  ROSE-G RNA thermometer. *A***, replicate of in-line probing of 3′-end labeled wild-type (left) and G32C mutant (right) *SB* RNA thermometer. ROSE-G and SD regions are boxed. Secondary structure features labeled with same colors as Figure 4B. ***B***, in-line probing resolved gels band intensity quantification at 42 °C of both Shine-Dalgarno (SD) and ROSE-G regions normalized to loop 1 (L1) and A48 + A49 for both wild-type and G32C mutant.

**Supporting Table S1. ROSE-G RNA thermometer candidates found upstream of ABC transporter genes in bacteria.**

| **Species** | **Downstream Encoded Protein** | **P2 Sequence (5′ → 3′)^a^** |
| --- | --- | --- |
| *Algicella marina* | Dipeptide/oligopeptide/nickel ABC transporter ATP-binding protein | ***ACCTGCTGAGCGACGGCCTGAGGAGTGCCATG*** |
| *Alloyangia pacifica* | Dipeptide/oligopeptide/nickel ABC transporter ATP-binding protein | ***ACCTGCTGAGCGACGGCCTGAGGAGTGCGATG*** |
| *Atlantibacter hermannii* | Murein tripeptide/oligopeptide ABC transporter ATP binding protein OppF | ***GCCTGCTTTAAACCGGTGGAGGAACTGGTATG*** |
|  |  |  |
| *Atlantibacter subterraneus* |  |  |
|  |  |  |
| *Citrobacter freundii* |  |  |
|  |  |  |
| *Citrobacter cronae* |  |  |
|  |  |  |
| *Citrobacter werkmanii* |  |  |
|  |  |  |
| *Citrobacter europaeus* |  |  |
|  |  |  |
| *Citrobacter youngae* |  |  |
|  |  |  |
| *Citrobacter meridianamericanus* |  |  |
|  |  |  |
| *Citrobacter arsenatis* |  |  |
|  |  |  |
| *Citrobacter braakii* |  |  |
|  |  |  |
| *Citrobacter murliniae* |  |  |
|  |  |  |
| *Citrobacter pasteurii* |  |  |
|  |  |  |
| *Citrobacter tructae* |  |  |
| *Azospirillum sp.* | Branched-chain amino acid ABC transporter permease | ***GGCTGCTGGGCCGCAAGGAGGAGCGCACGGTATG*** |
| *Bradyrhizobium sp.* | Branched-chain amino acid ABC transporter permease | ***GCCTGCTCGGCCGCGCCGAGGAGCGCACCGTATG*** |
|  |  |  |
| *Bradyrhizobium genosp.* |  |  |
| *Bradyrhizobium diazoefficiens* | Branched-chain amino acid ABC transporter permease | ***GACTGCTCGGCCGCGCCGAGGAGCGCACGGTATG*** |
| *Bradyrhizobium ottawaense* |  |  |
| *Bradyrhizobium guangdongense* |  |  |
| *Bradyrhizobium arachidis* |  |  |
| *Bradyrhizobium guangzhouense* |  |  |
| *Bradyrhizobium oligotrophicum* | Putative branched-chain amino acid ABC transporter permease protein | ***GCCTGCTCGGCCGAGCCGAGGAGCGCACGGTATG*** |
| *Buttiauxella agrestis* | Oligopeptide ABC transporter ATP binding protein OppF | ***GCCTGCTTTAAGCCAGTGGAGGAATTAGTATG*** |
| *Cronobacter dublinensis* | Cysteine/glutathione ABC transporter ATP-binding protein/permease | ***CACTGCTGGCGCACCGTCAGGAGGAGATTTAAATG*** |
|  |  |  |
| *Cronobacter condimenti* |  |  |
| *Citrobacter freundii* | Murein tripeptide/oligopeptide ABC transporter ATP binding protein OppF | ***GCCTGCTTTAAGCCGGTGGAGGAACTGGTATG*** |
|  |  |  |
| *Citrobacter portucalensis* |  |  |
|  |  |  |
| *Citrobacter bitternis* |  |  |
|  |  |  |
| *Enterobacteriaceae bacterium* |  |  |
| *Enterobacter kobei* |  |  |
| *Enterobacter sp.* |  |  |
|  |  |  |
| *Metakosakonia sp.* |  |  |
|  |  |  |
| *Phytobacter diazotrophicus* |  |  |
|  |  |  |
| *Phytobacter ursingii* |  |  |
|  |  |  |
|  |  |  |
| *Superficieibacter sp.* |  |  |
|  |  |  |
| *Shigella flexneri* |  |  |
|  |  |  |
|  |  |  |
| *Citrobacter freundii* | Murein tripeptide/oligopeptide ABC transporter ATP binding protein OppF | ***GCCTGCTTTAAACCGGTGGAGGATCTGGCATG*** |
|  |  |  |
| *Citrobacter amalonaticus* |  |  |
|  |  |  |
| *Citrobacter farmeri* |  |  |
|  |  |  |
| *Citrobacter telavivensis* |  |  |
| *Citrobacter koseri* | Murein tripeptide/oligopeptide ABC transporter ATP binding protein OppF | ***GCCTGCTTTAAACCGGTGGAGGAGCTGGTATG*** |
|  |  |  |
| *Citrobacter sedlakii* |  |  |
| *Citrobacter portucalensis* | Murein tripeptide/oligopeptide ABC transporter ATP binding protein OppF | ***GCCTGCTTTAAGCCGGTGGAGGAATTGGTATG*** |
|  |  |  |
| *Kosakonia calanthes* |  |  |
|  |  |  |
| *Paramixta manurensis* |  |  |
| *Citrobacter rodentium* | Murein tripeptide/oligopeptide ABC transporter ATP binding protein OppF | ***GCCTGCTTTAAACCGGTGGAGGATCTGGTATG*** |
| *Cronobacter sakazakii* | Cysteine/glutathione ABC transporter ATP-binding protein/permease | ***CGCTGCTGGCGCACCGTCAGGAGGAGATTTAAATG*** |
|  |  |  |
| *Cronobacter muytjensii* |  |  |
|  |  |  |
| *Cronobacter turicensis* |  |  |
|  |  |  |
| *Cronobacter malonaticus* |  |  |
|  |  |  |
| *Cronobacter universalis* |  |  |
| *Duffyella gerundensis* | Oligopeptide ABC transporter ATP binding protein OppF | ***GCCTGCTTTAAGCCGGTTGAGGAGTTAGTATG*** |
|  |  |  |
| *Erwinia sp.* |  |  |
|  |  |  |
| *Enterobacteriaceae bacterium* | Oligopeptide/dipeptide ABC transporter, ATP-binding protein | ***GCCTGCTTTAAGCCTGTGGGGGAACTGGTATG*** |
| *Pseudocitrobacter corydidari* | ABC transporter ATP binding protein btuD |  |
| *Enterobacter hormaechei* | Murein tripeptide/oligopeptide ABC transporter ATP binding protein OppF | ***GCCTGCTTTAAGCCGCTGGAGGAGCTGGTATG*** |
|  |  |  |
| *Enterobacter cloacae* |  |  |
|  |  |  |
| *Enterobacter intestinihominis* |  |  |
| *Erwinia aphidicola* | Murein tripeptide/oligopeptide ABC transporter ATP binding protein OppF | ***GCCTGCTTTAAGCCCGTGGAGGAGTTGGTATG*** |
| *Ewingella americana* | ABC transporter ATP-binding protein | ***GCCTGCTTTAAGCCGGTTGAGGAGTTGGTATG*** |
| *Erwinia amylovora* | Murein tripeptide/oligopeptide ABC transporter ATP binding protein OppF | ***GCCTGCTTTAAACCCGTGGAGGAGCTGGTATG*** |
|  |  |  |
| *Erwinia billingiae* |  |  |
| *Erwinia persicina* | Murein tripeptide/oligopeptide ABC transporter ATP binding protein OppF | ***GCCTGCTTTAAGCCCGTGGAGGAACTGGTATG*** |
| *Erwinia rhapontici* | Oligopeptide ABC transporter ATP binding protein OppF | ***GCCTGCTTTAAGCCCGTGGAGGCGTTGGTATG*** |
| *Escherichia coli* | Murein tripeptide/oligopeptide ABC transporter ATP binding protein OppF | ***GCCTGCTTTAAACCGGTGGAGGAGCTGTTATG*** |
| *Klebsiella aerogenes* | Murein tripeptide/oligopeptide ABC transporter ATP binding protein OppF | ***GCCTGCTTTAAACCGGTGGGGGACCTGCTATG*** |
| *Klebsiella huaxiensis* | Cysteine/glutathione ABC transporter ATP-binding protein/permease | ***CCCTGCTCGCACACCGTCAGGAGGAGATTTAAATG*** |
|  |  |  |
| *Klebsiella michiganensis* | Cysteine/glutathione ABC transporter ATP-binding protein/permease | ***CCCTGCTCGCTCATCGTCAGGAGGAGATTTAAATG*** |
| *Pseudescherichia vulneris* |  |  |
| *Klebsiella pneumoniae* | Murein tripeptide/oligopeptide ABC transporter ATP binding protein OppF+A114:B124 | ***GCCTGCTTTAAACCGGTGGGGGATCTGCTATG*** |
|  |  |  |
| *Klebsiella quasipneumoniae* |  |  |
|  |  |  |
| *Klebsiella variicola* |  |  |
|  |  |  |
| *Klebsiella quasivariicola* |  |  |
|  |  |  |
| *Klebsiella africana* |  |  |
|  |  |  |
| *Yokenella regensburgei* |  |  |
| *Kosakonia cowanii* | Murein tripeptide/oligopeptide ABC transporter ATP binding protein OppF | ***GCCTGCTTTAAGCCGGTGGAGGAGTTAGTATG*** |
|  |  |  |
| *Pantoea sp.* |  |  |
|  |  |  |
| *Pantoea agglomerans* |  |  |
| *Pantoea alfalfae* |  |  |
|  |  |  |
| *Pantoea anthophila* |  |  |
|  |  |  |
| *Pantoea eucalypti* |  |  |
|  |  |  |
| *Pantoea piersonii* |  |  |
|  |  |  |
| *Pantoea vagans* |  |  |
|  |  |  |
| *Siccibacter turicensis* |  |  |
|  |  |  |
| *Kosakonia cowanii* | Murein tripeptide/oligopeptide ABC transporter ATP binding protein OppF | ***GCCTGCTTTAAGCCGGTGGAGGAGCTAGTATG*** |
| *Loktanella salsilacus* | ATP-binding cassette domain-containing protein | ***ACCTGCTCAGCGACGGTCTAAGGAGCGCGATG*** |
| *Mixta calida* | Murein tripeptide/oligopeptide ABC transporter ATP binding protein OppF | ***GCCTGCTTTAAGCCGGTGGAGGAATTAGTATG*** |
|  |  |  |
| *Mixta gaviniae* |  |  |
|  |  |  |
| *Mixta hanseatica* |  |  |
| *Pantoea agglomerans* |  |  |
| *Pantoea alhagi* |  |  |
|  |  |  |
| *Pantoea cypripedii* |  |  |
|  |  |  |
|  |  |  |
| *Mixta intestinalis* | Oligopeptide ABC transporter ATP binding protein OppF | ***GCCTGCTTTAAGCCGGTGGAGGAATTAGCATG*** |
| *Pantoea alhagi* | Murein tripeptide/oligopeptide ABC transporter ATP binding protein OppF | ***GCCTGCTTTAAGCCGGTGGAGGAGTTGGTATG*** |
|  |  |  |
| *Winslowiella toletana* |  |  |
| *Paracoccus sp.* | ATP-binding cassette domain-containing protein | ***ACCTGCTCAGCGACGGTCTGAGGAGTGCGATG*** |
| *Paracoccus marcusii* | ATP-binding cassette domain-containing protein | ***ATCTGCTCAGCGACGGTCTGAGGAGTGCGATG*** |
| *Providencia hangzhouensis* | Murein tripeptide/oligopeptide ABC transporter ATP binding protein OppF | ***GCCTGCTTTAAGCCACTGGAGGAATTGGTATG*** |
|  |  |  |
| *Providencia huaxiensis* |  |  |
|  |  |  |
| *Providencia rettgeri* |  |  |
| *Providencia rustigianii* | Glutathione import ATP-binding protein | ***GCCTGCTTTAAGCCTGTGGAGGAGTTAGTATG*** |
|  |  |  |
| *Providencia rustigianii JH-1* | Oligopeptide ABC transporter ATP-binding protein OppF |  |
| *Pseudescherichia vulneris* | Murein tripeptide/oligopeptide ABC transporter ATP binding protein OppF | ***GCCTGCTTTAAGCCGGTGGAGGAGCTGGTATG*** |
| *Rhodopseudomonas boonkerdii* | Branched-chain amino acid ABC transporter permease | ***GCCTGCTCGGCCGTCCCGAGGAGCGCACCGTATG*** |
| *Rhodovulum sulfidophilum* | Dipeptide/oligopeptide/nickel ABC transporter ATP-binding protein | ***ACCTGCTGAGCGACGGCGTGAGGAGTGCGATG*** |
| *Scandinavium goeteborgense* | Murein tripeptide/oligopeptide ABC transporter ATP binding protein OppF | ***GCCTGCTTTAAACCGGTGGGGGATCTGGTATG*** |
| *Sagittula stellata E-37* | ABC transporter ATP-binding protein | ***ACCTGCTGAGCGACGGGCTGAGGAGTGCGATG*** |
| *Sagittula sp.* | Dipeptide/oligopeptide/nickel ABC transporter ATP-binding protein |  |
| *Serratia liquefaciens* | ABC transporter ATP-binding protein (Likely OppF) | ***GCCTGCTTTAAACCAGTGGAGGCGTTGGTATG*** |
| *Variovorax boronicumulans* | ABC transporter substrate-binding protein | ***CCCTGCTTTCCCTGAAGGAGAAGAACATG*** |
| *Variovorax paradoxus* | ABC transporter glutamine-binding protein GlnH | ***CCCTGCTTTCCATGAAGGAGAAGAACATG*** |
| *Yersinia alsatica* | Murein tripeptide/oligopeptide ABC transporter ATP binding protein OppF | ***GCCTGCTATAAGCCAGTAGGGGAATTGGTATG*** |
|  |  |  |
| *Yersinia kristensenii* |  |  |
|  |  |  |
| *Yersinia rochesterensis* |  |  |
| *Yersinia intermedia* | Murein tripeptide/oligopeptide ABC transporter ATP binding protein OppF | ***GCCTGCTATAAGCCAGTGGGGGAATTAGTATG*** |

^a^ Segment coding the ROSE-G motif underlined.

**Supporting Table S2. Sequences of oligonucleotides used in this study.**

| **Oligonucleotide** | **Description** | **Sequence** (5′ to 3′) |
| --- | --- | --- |
| *SB ^a^* | Sequence for β-galactosidase assay, *in vitro* translation, and in-line probing | ATCCGAGGTTTTATTGATATGGTCTCGCCCTGCTTTCCCTGAAGGAGAAGAACATG |
| *SB* – P2*^a^* | Sequence for β-galactosidase assay | CCCTGCTTTCCCTGAAGGAGAAGAACATG |
| *cydC^a^ (Cronobacter sakazakii)* | Sequence for β-galactosidase assay | CGAGGCCGATGGTTATTTCGCGGCGCTGCTGGCGCACCGTCAGGAGGAGATTTAAATG |
| *cydC^a^*  *(Klebsiella michiganensis)* | Sequence for β-galactosidase assay | AGCTGGCGGCCGCTGGCGGCCCCTTCGCGACCCTGCTCGCTCATCGTCAGGAGGAGATTTAAATG |
| *oppF^a^* | Sequence for β-galactosidase assay | GCCTGCTTTAAACCGGTGGAGGAGCTGTTATG |
| G32C*^a^* | Sequence for β-galactosidase assay and *in vitro* translation and in-line probing | ATCCGAGGTTTTATTGATATGGTCTCGCCCTCCTTTCCCTGAAGGAGAAGAACATG |
| U36C*^a^* | Sequence for β-galactosidase assay | ATCCGAGGTTTTATTGATATGGTCTCGCCCTGCTTCCCCTGAAGGAGAAGAACATG |
| G32C, ∆U36 *^a^* | Sequence for β-galactosidase assay and *in vitro* translation | ATCCGAGGTTTTATTGATATGGTCTCGCCCTCCTTCCCTGAAGGAGAAGAACATG |
| G32C, U36C *^a^* | Sequence for β-galactosidase assay and in-line probing | ATCCGAGGTTTTATTGATATGGTCTCGCCCTCCTTCCCCTGAAGGAGAAGAACATG |

*^a^* underlined ATG is the start codon for *bgaB*
